# A novel ATAC-seq approach reveals lineage-specific reinforcement of the open chromatin landscape via cooperation between BAF and p63

**DOI:** 10.1186/s13059-015-0840-9

**Published:** 2015-12-18

**Authors:** Xiaomin Bao, Adam J. Rubin, Kun Qu, Jiajing Zhang, Paul G. Giresi, Howard Y. Chang, Paul A. Khavari

**Affiliations:** Program in Epithelial Biology, Stanford University, 269 Campus Drive, Stanford, CA 94305 USA; Howard Hughes Medical Institute, Stanford University, Stanford, CA 94305 USA; Veterans Affairs Palo Alto Healthcare System, 3801 Miranda Ave, Palo Alto, CA 94304 USA

**Keywords:** BAF, Differentiation, Nucleosomes, Open chromatin, p63

## Abstract

**Background:**

Open chromatin regions are correlated with active regulatory elements in development and are dysregulated in diseases. The BAF (SWI/SNF) complex is essential for development, and has been demonstrated to remodel reconstituted chromatin in vitro and to control the accessibility of a few individual regions *in vivo*. However, it remains unclear where and how BAF controls the open chromatin landscape to regulate developmental processes, such as human epidermal differentiation.

**Results:**

Using a novel “on-plate” ATAC-sequencing approach for profiling open chromatin landscapes with a low number of adherent cells, we demonstrate that the BAF complex is essential for maintaining 11.6 % of open chromatin regions in epidermal differentiation. These BAF-dependent open chromatin regions are highly cell-type-specific and are strongly enriched for binding sites for p63, a master epidermal transcription factor. The DNA sequences of p63 binding sites intrinsically favor nucleosome formation and are inaccessible in other cell types without p63 to prevent ectopic activation. In epidermal cells, BAF and p63 mutually recruit each other to maintain 14,853 open chromatin regions. We further demonstrate that BAF and p63 cooperatively position nucleosomes away from p63 binding sites and recruit transcriptional machinery to control tissue differentiation.

**Conclusions:**

BAF displays high specificity in controlling the open chromatin landscape during epidermal differentiation by cooperating with the master transcription factor p63 to maintain lineage-specific open chromatin regions.

**Electronic supplementary material:**

The online version of this article (doi:10.1186/s13059-015-0840-9) contains supplementary material, which is available to authorized users.

## Background

Somatic tissue differentiation is an essential process that restricts cellular potential and enhances cell fate for specialized functions, such as the barrier function of epidermal tissue. Failure to engage tissue-specific differentiation underlies a spectrum of human diseases, including lipodystrophies [1], psoriasis [2], and cancers [3, 4]. It remains incompletely understood, however, how cell fate is determined and is restricted to the differentiation state.

An increasingly appreciated correlate of cell fate is the cell-type-specific open chromatin landscape [5], as defined by the specific regions of chromatin exhibiting high DNA accessibility in a given cell or tissue type. These open chromatin regions span an average of 300 base pairs [6], comprise 1–3 % of the total genome [7], and can be identified by DNase I hypersensitive sites sequencing (DNase-seq) [8] or formaldehyde-assisted isolation of regulatory elements sequencing (FAIRE-seq) [9]. Comparison of open chromatin landscapes across different cell types has revealed distinct patterns. In contrast to open sites that are widely shared across diverse cell types, which can be near commonly active transcription start sites (TSSs) of housekeeping genes [7], many cell-type-specific open chromatin regions are intergenic and may correspond to tissue-specific regulatory regions.

The BAF chromatin remodeling complex, composed of one catalytic subunit (BRG1 or BRM) and 10 regulatory subunits, is essential for the differentiation of multiple somatic tissues such as muscle, fat, blood, and epidermis [10–15]. BAF is also the most frequently mutated chromatin-modifying complex in all human cancers [16, 17]. Using in vitro reconstituted chromatin and at only selected genomic loci in vivo, the BAF complex has been demonstrated to alter DNase I footprinting and to facilitate the DNA binding of a variety of transcription factors (TFs), including zinc-finger, helix-loop-helix, and rel-domain containing proteins [11, 18, 19]. However, it remains unclear how BAF controls the open chromatin landscape on a genome-wide scale, and whether BAF can systematically prioritize its interaction with specific TFs in a given developmental process such as tissue differentiation.

There are several technical barriers to understanding the control of open chromatin by BAF on a genome-wide scale. First, the BAF-dependent open chromatin regions cannot be simply represented by its chromatin immunoprecipitation sequencing (ChIP-seq) data, because the chromatin binding of the BAF complex can be mediated by its non-catalytic subunits in addition to the catalytic subunits, and because many BAF binding sites are also controlled redundantly by CHD and ISWI chromatin remodelers [20]. Second, both DNase-seq and FAIRE-seq typically require tens of millions of cells [6, 7]. This amount of input material is more challenging in the context of BAF loss, which impairs the proliferation of many cell types [21]. Lastly, the two BAF catalytic subunits BRG1 and BRM have been demonstrated to share redundant functions in somatic tissue development [12, 22]. However, limited genomic characterizations so far have only been attempted in the context of altered Brg1 function alone. By expressing a dominant-negative Brg1 mutant, only 1.9 % DNase hypersensitive sites (DHSs) were lost in the 3134 mouse mammary epithelial cell line [20]. In mouse CD36^+^ cells, knocking down Brg1 alone also only modestly affected the nucleosome positioning at GATA1 sites without affecting GATA1 genomic binding [14]. It remains unclear, however, how the open chromatin landscape could be altered with the loss of both BRG1 and BRM.

Here we address the role of the BAF complex in controlling the genome-wide open chromatin landscape using a model of human epidermal differentiation. We and others have demonstrated previously that loss of the BAF catalytic subunits BRG1 and BRM abolished epidermal tissue differentiation [12, 13], yet the mechanism underlying how BAF controls epidermal differentiation remains unclear. To characterize the open chromatin landscape in the context of BAF loss, we leveraged the recent development of the assay for transposase-accessible chromatin with high-throughput sequencing (ATAC-seq), which requires only 50,000 cells as input material [23]. Because the original protocol was optimized for suspension cells, we further innovated a novel “on-plate ATAC-seq” method, which allows direct probing of open chromatin regions in adherent epidermal cells growing on a 96-well plate, bypassing the disruptive trypsinization process [24, 25]. Our high-quality data produced using this on-plate ATAC-seq method allowed exploration of the impact of the BAF complex on the open chromatin landscape during epidermal differentiation.

By characterizing the open chromatin landscape in the context of depleting both BAF catalytic subunits BRG1 and BRM, here we show that BAF is required to maintain 17,617 open chromatin sites (11.6 %) during epidermal cell differentiation, despite the fact that BAF binding is enriched in 50.3 % of open chromatin regions. BAF-dependent open chromatin regions are strikingly epidermal cell-type specific. They are also strongly enriched for binding sites of the epidermal master TF p63. p63 binding elements are enriched in ectodermal-derived tissue types [5]. In keratinocytes, p63 binding is enriched in enhancers, and has a strong correlation with the histone modification H3K4me1 [26]. However, it remains incompletely understood how p63 binding to DNA is facilitated in the context of chromatin. We show that both BAF and p63 co-regulate the expression of 236 epidermal differentiation genes, that they mutually reinforce each other’s binding, and that they are both required for sustaining open chromatin formation at p63 binding sites. Thus, these results demonstrate a new mode of cooperative action between BAF and p63 in controlling the keratinocyte-specific open chromatin landscape to enable epidermal differentiation. These data suggest that tissue terminal differentiation requires cooperation of highly selective BAF chromatin remodeling activity and a lineage-specific TF to sustain a tissue-specific open chromatin landscape.

## Results

### BAF catalytic subunits are required to maintain open chromatin regions

The catalytic subunits of the BAF chromatin remodeling complex BRG1/BRM are essential for epidermal differentiation [12, 13]. We first reconfirmed this in primary human keratinocyte (NHEK) culture in vitro, where loss of BRG1/BRM strongly inhibited the induction of differentiation genes, such as *KRT1*, *KRT10*, and *LCE3D* (Additional file 1: Figure S1a). During the time course of 4-day keratinocyte differentiation, KRT1 expression was strongly induced in the control condition but not with BRG1/BRM loss (Additional file 1: Figure S1c). Differentiation day 4 was used as the time point for comparative genomic characterization in this study. RNA-seq analysis indicated that the gene set controlled by BAF is highly enriched with Gene Ontology (GO) terms such as “epidermis development” and “epidermal cell differentiation” (Additional file 1: Figure S1b; Additional file 2: Table S1). To interrogate the role of BAF in genome accessibility during differentiation, we used ATAC-seq to assess genome-wide accessibility to the Tn5 transposase, which acts in this context similarly to DNase I [23], in normal cells and in cells depleted of BRG1/BRM to remove functional catalytic subunits of the BAF complex (BAFi).

**Fig. 1:**
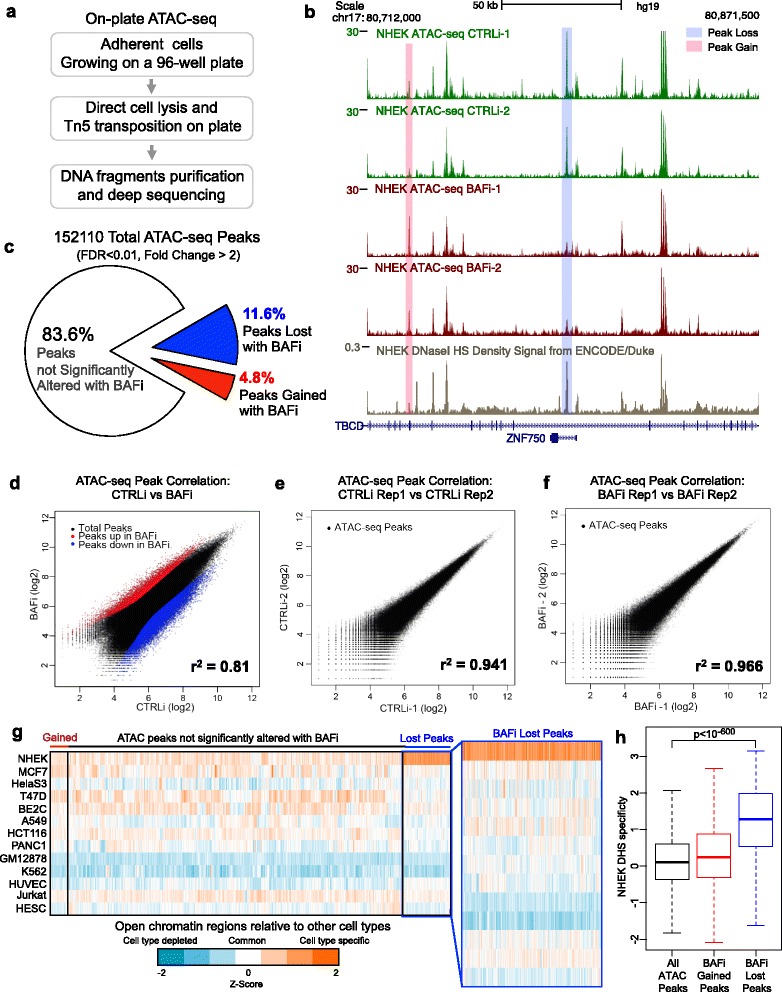
BAF controls the open chromatin landscape in epidermal differentiation. **a** Workflow of on-plate ATAC-seq analysis in control (*CTRLi*) and BRG1/BRM depletion (*BAFi*) conditions in primary human keratinocyte differentiation. **b** UCSC genome browser tracks showing replicates of ATAC-seq, generated from individual wells of differentiating human keratinocytes growing on a 96-well plate, in control (*green tracks*, n = 2) and BAF loss (*red tracks*, n = 2) conditions at the *ZNF750* TF locus. Published DNase-seq in normal human epidermal keratinocyte (*NHEK*) cells from publically available ENCODE data (*beige track*) is also included as reference. An example of a decreased peak with BAF loss is shaded in *blue*, and an example of an increased peak is shaded in *red*. **c** Pie chart showing the distribution of the total 152,110 ATAC-seq peaks relative to gain and loss with BAFi. **d**–**f** Scatter plots showing the ATAC-Seq signal correlation between BAFi versus CTRLi, as well as between technical replicates. **g** Heatmap showing the cell-type specificity of both BAF-dependent and BAF-independent ATAC-seq peaks, as compared to DNase hypersensitive sites (*DHS*) open chromatin accessibility in 14 representative cell lines. **h** Boxplot showing the z-score distribution of BAF-dependent ATAC-seq peaks, as compared to all and gained ATAC-seq peaks. The lost ATAC-seq peaks with BAFi are highly enriched with keratinocyte-specific open chromatin regions (*p* < 10^-600^, Kolmogorov–Smirnov test). *TF* transcription factor, *ENCODE* encyclopedia of DNA elements.

To adapt ATAC-seq to the adherent human keratinocytes with minimal perturbation to living cells, we developed an on-plate ATAC-seq technique. In this approach, we directly applied Tn5 transposase to cells growing on a 96-well plate, after a short permeabilization step. We then purified the fragmented genomic DNA from each well “on plate” for deep sequencing and data analysis (Fig. 1a). This on-plate technique generated sequencing libraries with open chromatin accessibility information comparable to DNase-seq libraries (ENCODE), but used only 0.1 % of input cell numbers and bypassed the need for trypsinization (Fig. 1b). Observed open chromatin sites were highly enriched in putative distal regulatory regions (Additional file 1: Figure S1d–f). The two replicates in both control and knockdown conditions were highly correlated to each other (r^2^ = 0.941, 0.966. Fig. 1b,d–f), indicating strong data reproducibility. Regions of both gained and decreased genome accessibility were observed with functional BAF loss (Fig. 1b,d). In total, the BAF complex is required to maintain ~11.6 % of total open chromatin regions in epidermal differentiation. A much smaller fraction of the genome (4.8 %) displayed increased accessibility with BAFi (false discovery rate [FDR] < 0.01, fold change >2, Fig. 1c). This is consistent with the notion that the BAF complex functions more often as a transcription activator for gene expression in mammals.

Because open chromatin regions comprise both cell-type-specific regions as well as common regions, we first compared both BAF-dependent and independent ATAC regions with the ENCODE DNase-seq open chromatin regions from other cell types. While the gained regions as well as the BAF-independent regions did not display strong cell-type specificity, the lost peaks with BAFi were significantly enriched with keratinocyte-specific open chromatin regions (*p* < 10^−600^; Fig. 1g, h). These data suggest that BAF is essential to maintain the cell-type-specific open chromatin landscape during epidermal differentiation.

### BAF is required to maintain open chromatin regions that bind p63

**Fig. 2:**
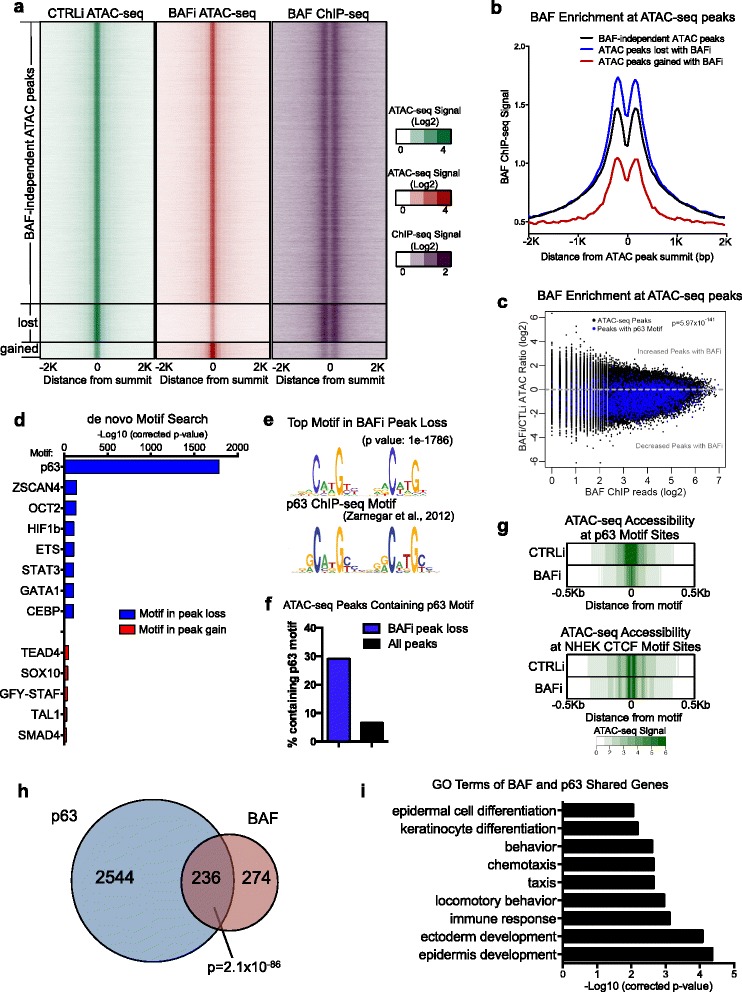
BAF is enriched at and promotes open chromatin sites with p63 binding. **a** ATAC-seq summit-centered heatmap of ATAC-seq signal in control (*CTRLi*) and BAF-loss (*BAFi*) conditions, as well as BAF ChIP-seq signal in the same regions for all ATAC-seq peaks. The ATAC-seq peaks are sorted in BAF-independent, lost, and gained groups. **b** Average diagram of BAF ChIP-seq signal at BAF-independent, lost, and gained open chromatin regions. **c** Scatter plot demonstrating the relative enrichment of BAF binding at gained/lost ATAC-seq peaks. The p63-motif containing ATAC-seq peaks are highlighted in *blue*. **d** Bar graph showing the − Log10 *p*-value of TF motifs identified from a de novo Homer motif search, based on the ATAC-seq peaks gained or lost with BAFi. **e** Comparison of top motifs identified from the ATAC-seq analysis with a known p63 motif (weighted matrix). **f** Bar graph showing the percentage of ATAC-seq peaks with the p63 motif in both total ATAC-seq peaks as well as in BAFi peak loss. **g** Average diagram of genome-wide open chromatin accessibility at p63 and CTCF regions comparing CTRLi and BAFi conditions. **h** Venn diagram showing the overlap of gene sets controlled by p63 and BAF in keratinocyte differentiation. **i** Gene Ontology analysis of the 236 genes shared by BAF and p63. *bp* base pair, *kb* kilobase pair, *TF* transcription factor

Previous studies indicate that BAF binds broadly on chromatin, and the binding sites are divergent in various cell types [27, 28]. To test whether BAF binds directly to these BAF-dependent open chromatin regions, as identified from our ATAC-seq analysis, we first performed BAF ChIP-seq in keratinocytes using an antibody that recognizes both BAF catalytic subunits BRG1 and BRM [29]. BAF binding was correlated with the open chromatin regions detected using ATAC-seq in normal differentiating keratinocytes (Fig. 2a; Additional file 1: Figure S2a). Furthermore, BAF binding was more enriched in the ATAC-seq peaks that were decreased with BAFi, but not in the gained peaks (Fig. 2a–c). These data suggest that BAF functions to directly maintain a subset of open chromatin regions where BAF binding is highly enriched.

To further characterize these BAF-dependent open chromatin regions, we performed de novo motif enrichment analysis on the 17,617 open chromatin regions that were lost with BAFi and the 7374 regions that gained ATAC-seq peaks with BAFi. The top motif enriched in BAF-dependent open chromatin corresponded to the p63 motif based on p63 ChIP-seq data (Fig. 2d–f) [30, 31]. p63 is the lineage-specifying TF essential for epidermal development as well as the TF required post-natally to maintain stratified epithelial tissue identity and terminal differentiation [32, 33]. Other p53 family member TFs, such as p53 and p73, were also expressed in epidermal cells; however, p63 was expressed at higher levels than these other two genes (Additional file 1: Figure S2b). p63 motif enrichment was striking (*p* = 1 × 10^−1786^), but only in genomic regions that lost openness with BAFi; it was not enriched at sites that gained accessibility with BAFi (Fig. 2d). Further analysis of ATAC-seq accessibility at individual p63 motif sites in p63 binding sites indicated that 21.1 % of these sites became inaccessible after BAF loss (Additional file 1: Figure S2c). Binding regions for 808 other TFs that are expressed at RPKM > 1, as measured by RNA-seq (Additional file 1: Figure S2d), including many that are functionally important in epidermal differentiation, such as *HOPX1*, *CTNNB1*, *FOSL2*, *ZNF750*, *GRHL3*, *YAP1*, *RBPJ*, *OVOL1*, *MAF*, *MAFB*, *CEBPA*, *RARG,* and *KLF4*, did not display such enrichment (Fig. 2d). ATAC-seq analysis further demonstrated a selective loss of accessibility at p63 motif regions with BAF depletion, but not at CTCF motif regions (Fig. 2g). In addition, ATAC-seq accessibility at KLF4 motif regions was not significantly altered at KLF4 motif regions (Additional file 1: Figure S2e). These data suggest that BAF maintains open chromatin regions with a strong selectivity for p63 binding in epidermal differentiation.

The dependence on BAF to maintain open chromatin regions containing p63 binding sites suggested that BAF and p63 may cooperate to control the expression of epidermal differentiation genes. To test this, we compared the target gene sets of BAF and p63 using RNA-seq. This demonstrated that BAF and p63-dependent target genes (fold change > 3 with depletion, FDR < 0.01) displayed highly significant overlap (*p* = 2.1 × 10^−86^), with 236 genes controlled jointly by both BAF and p63 (Fig. 2h; Additional file 1: Figure S2f, g; Additional file 2: Table S2). The GO terms for this shared target gene set were enriched with “epidermis development” (*p* = 4.8 × 10^−5^) and “epidermal cell differentiation” (*p* = 8.7 × 10^−3^) (Fig. 2i). Many of these shared genes were suppressed or induced in both BAFi and p63i conditions (Additional file 1: Figure S2e, f), suggesting that BAF and p63 may co-regulate epidermal tissue differentiation in a process that involves BAF-dependent open chromatin regions enriched for p63 binding.

### BAF regulates nucleosome positioning and recruits transcriptional machinery

**Fig. 3:**
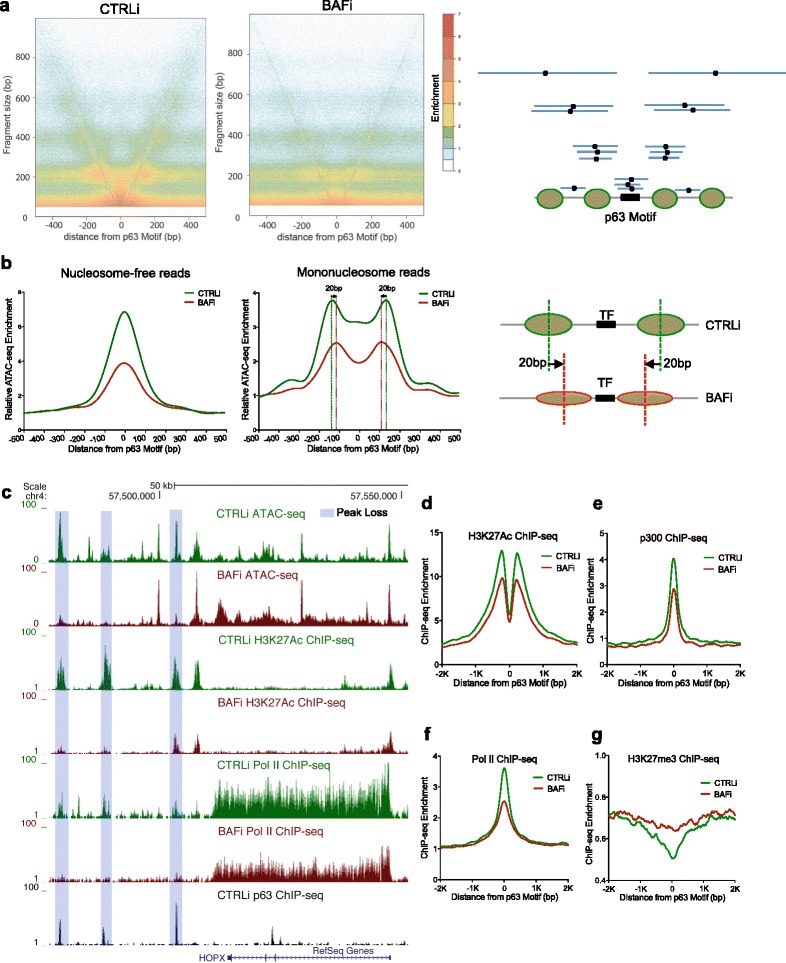
BAF regulates nucleosome positioning and recruits transcriptional machinery. **a** V-plot analysis of ATAC-seq fragments near p63 motif regions in both control (*CTRLi*) and BAF loss (*BAFi*) conditions. Schematic illustration of the V-plot analysis with phased nucleosome is included on the right. **b** Average diagram of nucleosome-free fragments (<100 bp) and mononucleosome fragment (180–247 bp) at p63 motif sites, demonstrating an average compaction of 40 bp at p63 sites with BAF loss. **c** UCSC genome browser tracks comparing ATAC-seq, H3K27Ac ChIP-seq, and Pol II ChIP-seq between CTRLi and BAFi, relative to p63 ChIP-seq at the *HOPX* TF locus. Decreased peaks with BAF loss are shaded with *light blue*. **d–g** Genome-wide average diagram of ChIP-seq (p63, H3K27Ac, Pol II, p300, and H3K27me3) comparing control to BAF loss. *bp* base pair, *kb* kilobase pair, *TF* transcription factor

Because BAF ATPase activity has been demonstrated to directly regulate nucleosome positioning on DNA templates in vitro [34, 35], we next tested whether BAF also controls nucleosome positioning in living cells at p63 motif sites across the genome. Owing to the physical hindrance by nucleosomes, ATAC-seq transposition preferentially localizes to the inter-nucleosome regions [23]. Consistent with this, DNA fragments generated by pairs of transposases were enriched for sizes corresponding to nucleosome-free regions as well as mononucleosomes and dinucleosomes (Additional file 1: Figure S3a). We first visualized nucleosome positioning without any bias towards fragment size using V-plot analysis, which shows the density of the midpoints relative to the p63 motif on the x-axis with the fragment separated by their length on the y-axis. In normal cells, positioned nucleosomes led to enriched fragment midpoints at the center of mononucleosome and dinucleosome positions surrounding p63 (Fig. 3a, left panel). This pattern was disordered with BAF loss at p63 sites (Fig. 3a, right panel) but not at CTCF sites (Additional file 1: Figure S3b), indicating that nucleosome positioning around p63 binding sites is disrupted in the absence of functional BAF.

Additionally, in normal differentiation, there was high accessibility at the p63 motif sites; however, this was also decreased with BAF loss. To quantify the changes observed in these two conditions, we used the nucleosome-free fragments (<100 base pairs [bp]) and mononucleosome fragments (180–247 bp), as published previously for nucleosome positioning analysis [23]. Consistent with the V-plot analysis, a decreased nucleosome-free signal was observed at p63 binding sites, indicating impaired accessibility at p63 motif regions and diminished nucleosome phasing adjacent to p63 motif with BAF loss. In addition, we found that the summits of the mononucleosome signals were shifted 20 bp towards the center of the p63 motif by BAF depletion (Fig. 3b), indicating an average of 40-bp nucleosome compaction at p63 motif regions in the absence of BAF. This altered nucleosome positioning with BAF loss was not universal, as no significant changes were observed at the CTCF motif (Additional file 1: Figure S3b-e). Therefore, nucleosome positioning analysis indicates that BAF is required to maintain genome accessibility at p63 binding motifs by positioning the nucleosomes away from the recognition sites.

We next hypothesized that BAF might help recruit transcriptional machinery and promote activation marks to sustain open chromatin at p63 binding regions. To test this, we performed ChIP-seq for the activating histone mark H3K27Ac, the histone acetyltransferase p300, RNA pol II, and the repressive histone mark H3K27me3, comparing control differentiating keratinocytes with BAF loss. This demonstrated that H3K27Ac, p300, and pol II levels were decreased with BAF loss, while H3K27me3 levels were increased (Fig. 3c–g). Therefore BAF is required for normal levels of p300, pol II, and H3K27Ac at p63 sites, where it is also required to oppose H3K27me3 and maintain open chromatin.

### p63 is also required to maintain open chromatin regions

**Fig. 4:**
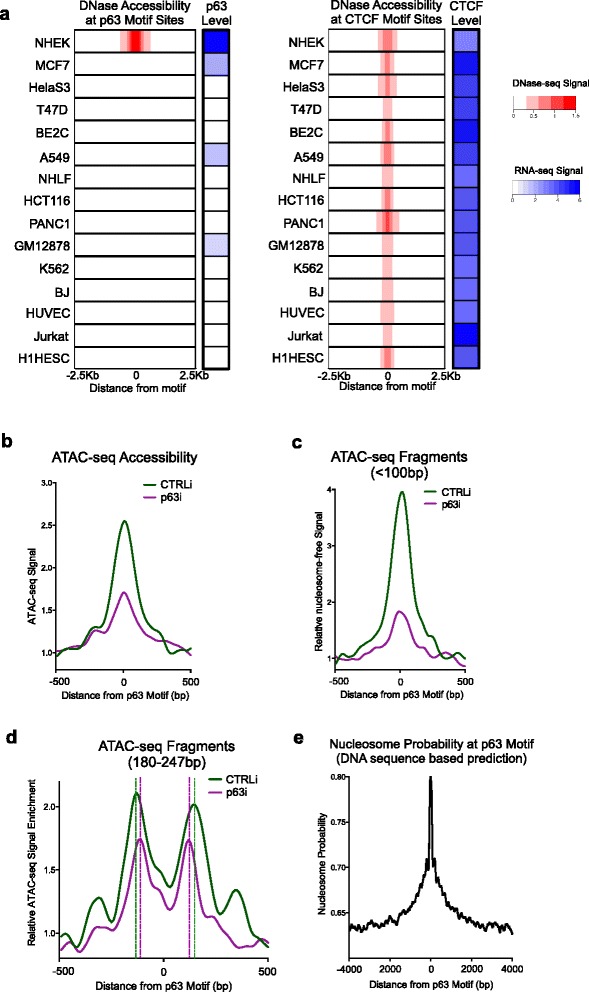
p63 is required to establish and to maintain open chromatin regions. **a** Heatmaps demonstrating chromatin accessibility at p63/CTCF motif sites in 15 different cell types in correlation with the p63/CTCF expression level. The accessibility to DNase I (ENCODE data) is demonstrated in *red*. RNA expression level (ENCODE data) of p63/CTCF in these cell types are include in parallel in *blue*. **b** Average diagram of total ATAC-seq signal at p63 sites comparing p63 loss (*p63i*) with control (*CTRLi*). **c** Average diagram of nucleosome-free fragments (<100 bp) at p63 motif regions, demonstrating p63 is required to maintain DNA accessibility at p63 motif regions. **d** Average diagram of mononucleosomal fragments (180–247 bp) comparing the nucleosome positioning at p63 sites between p63i and CTRLi conditions. Loss of p63 led to impaired nucleosome phasing and an average compaction of 25 bp at p63 motif sites. **e** Average diagram of predicted nucleosome binding probability based on DNA sequence composition in genomic regions centered by p63 motif, indicating that p63 motif sequences intrinsically favor nucleosome binding. *bp* base pair, *ENCODE* encyclopedia of DNA elements

As a lineage-determining TF, p63 expression is largely restricted to stratified epithelial tissues. To test the correlation between open chromatin and p63 expression, we examined 15 cell types from ectodermal, endodermal, and mesodermal origins, as well as embryonic stem cells, in ENCODE DNase-seq data. While these other cell types have comparable chromatin accessibility to the general TF CTCF, their p63 motif sites remain inaccessible to DNase I with the exception of human keratinocytes, where p63 is abundantly expressed (Fig. 4a), suggesting that p63 expression is required for open chromatin establishment at p63 sites. To test if p63 is essential to maintain open chromatin, we depleted p63 in differentiating human keratinocytes, and performed ATAC-seq analysis. Similar to BAF loss, p63 loss led to reduced accessibility, destabilized nucleosome phasing, and compacted nucleosome structure at the p63 motif sites within the p63 targets from ChIP-seq analysis (Fig. 4b–d). These data point to a p63 requirement for open chromatin maintenance at p63 binding sites.

Because local DNA sequence can directly impact nucleosome positioning, we calculated the intrinsic nucleosome binding probability based on genomic sequence [36, 37]. The genomic DNA sequences containing p63 motif sites displayed high nucleosome binding probability, which peaked at p63 motif sites with a probability of ~80 % (Fig. 4e). This high probability for nucleosome binding was absent in the shuffled genomic regions (Additional file 1: Figure S3f). These data suggest that the DNA sequences of p63 binding sites strongly favor nucleosome formation, and that these regions are intrinsically inaccessible in the absence of DNA binding proteins and chromatin remodelers. These data are consistent with our observation that both BAF and p63 are required to maintain open chromatin at p63 sites and support the existence of strong intrinsic suppressive mechanisms at these sites to prevent ectopic epithelial gene activation in non-epithelial cell types.

### BAF and p63 binding reinforce open chromatin

**Fig. 5:**
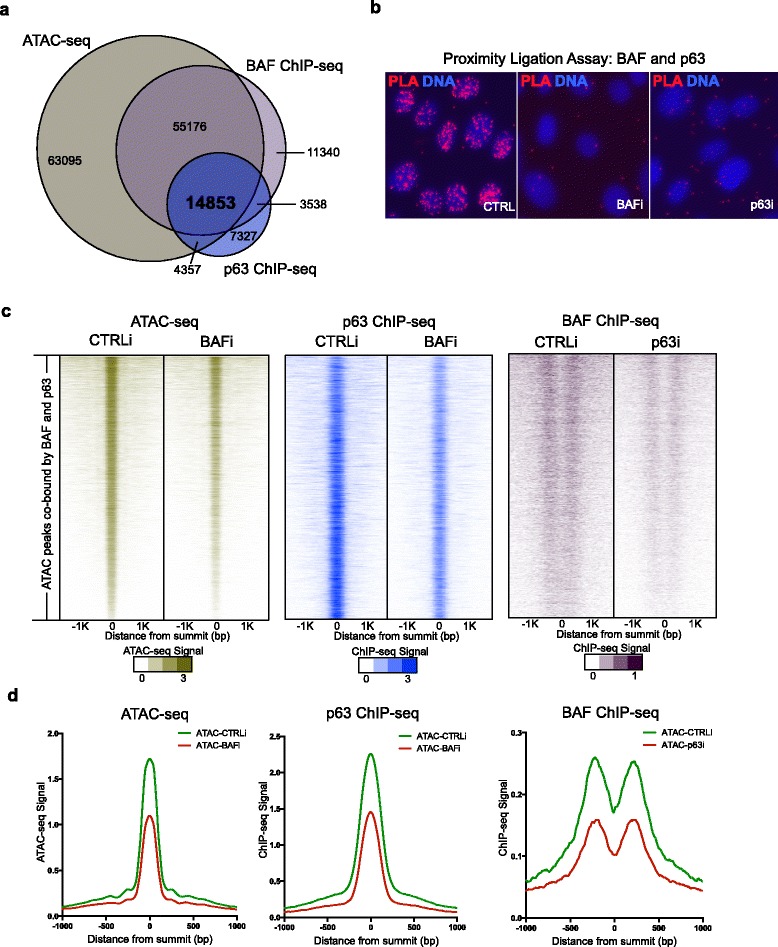
BAF and p63 cooperate in chromatin binding to reinforce open chromatin. **a** Venn diagram showing the overlap among ATAC-seq peaks, BAF ChIP-seq peaks, and p63 ChIP-seq peaks. **b** Proximity ligation analysis (*PLA*) in wild-type (*CTRL*, left), BAF-depleted (*BAFi*, middle), or p63-depleted (*p63i*, right) differentiating keratinocytes. *Red* represents PLA proximity signal for BAF and p63; *blue* is the Hoechst DNA stain. **c** Summit-centered heatmaps of 14,853 ATAC-seq peak regions co-bound with BAF and p63, showing ATAC-seq, p63 ChIP-seq, and BAF ChIP-seq signals in BAF or p63 loss and control conditions. **d** Average diagram of ATAC-seq, p63 ChIP-seq, and BAF ChIP-seq signals in BAF and p63 co-bound regions. *bp* base pair

The requirement of both BAF and p63 in controlling open chromatin suggests that BAF and p63 may co-localize to open chromatin regions. We compared the ChIP-seq peaks of BAF and p63 with ATAC-seq peaks in keratinocytes. p63 ChIP-seq peaks were called using MACS (version 1.4.2, *p*-value cutoff 1.00e − 05). Because BAF binds broadly on chromatin, BAF ChIP-seq peaks were called using the “broad” setting of MACS2 (threshold q 0.05). We observed a total of 14,853 open chromatin regions with co-binding of BAF and p63 (Fig. 5a). Using proximity ligation analysis, we further confirmed that BAF and p63 were in physical proximity to each other within the nucleus in differentiating human keratinocytes; this proximity signal was specific because it required the presence of both BAF and p63 (Fig. 5b).

Because BAF loss caused nucleosome compaction and decreased accessibility at p63 binding sites, we hypothesized that BAF could be essential for normal p63 binding across the genome. To test this, we first compared ATAC-seq accessibility at single nucleotide resolution at p63 and CTCF motif sites, as a method to predict the binding of these TFs. We observed a decreased signal for p63, but not CTCF with BAF loss (Additional file 1: Figure S4a, b), suggesting that p63 DNA binding is dependent on BAF. p63 levels did not change significantly with BAF loss (Additional file 1: Figure S1c), confirming that impaired p63 binding in this setting was not due to decreased p63 protein levels. To validate these findings, p63 ChIP-seq was performed in differentiating human keratinocytes with and without BAF depletion. p63 ChIP-seq peaks in both control and BAF-loss conditions were called using MACS (version 1.4.2, *p*-value cutoff 1.00e − 05). Out of a total of 30,075 normal control p63 binding peaks, 11,930 fell below the MACS peak-calling threshold with BAF loss. Even among the 18,145 peaks still called by MACS in both conditions, the p63 binding signal was reduced on average to half with BAF loss (Additional file 1: Figure S4c–e). In the 14,853 BAF and p63 co-bound ATAC-seq regions, BAF loss impaired both ATAC accessibility and p63 binding in these regions (Fig. 5c, d). These data indicate that normal p63 genomic binding requires intact BAF.

Prior work suggested that the BAF complex can itself be targeted by TFs to chromatin [19, 38]. To test whether p63 facilitates BAF binding in differentiating epidermal cells, we performed BAF ChIP-seq with p63 depletion. p63 loss correlated with reduced BAF binding at the p63 co-binding sites, but not at CTCF binding sites (Fig. 5c, d; Additional file 1: Figure S4f), indicating that p63 functions selectively on BAF association at p63 binding sites but not universally on BAF association with other sites.

Taken together, our data demonstrate that BAF maintains the epidermal cell-specific open chromatin landscape to promote tissue differentiation. In epithelial tissue, these BAF-dependent open chromatin regions are specifically enriched with p63 sites, which are intrinsically inaccessible to transcriptional machinery in a manner that may ensure repression of epithelial-specific gene expression in non-epithelial tissues that lack p63 expression. The chromatin remodeling activity of BAF controls nucleosome position to increase the accessibility of p63 binding sites, facilitating p63 genomic binding. The binding of p63, in turn, also enhances the chromatin targeting of BAF. This cooperation between BAF and p63 further enables local association of p300 and pol II, sustains activating histone marks, and opposes repressive histone marks in concert with promoting open chromatin formation and differentiation gene expression.

## Discussion

Here we present data indicating that the BAF complex and the epidermal lineage-determining TF p63 cooperate to remodel the open chromatin landscape and condition p63 binding regions for gene activation. In this setting, BAF chromatin remodeling catalytic subunits cooperate with p63 to control nucleosome positioning and to promote epidermal tissue-specific gene expression during differentiation. In this context, BAF is required to displace nucleosomes at an average of approximately 40 bp laterally away from p63 binding sites. This BAF-dependent genome accessibility at p63 sites is associated with enabled p63 binding, activating histone marks, RNA polymerase II machinery recruitment, and target gene expression. Epidermal tissue differentiation is a dynamic process that involves the repression of progenitor genes and the induction of differentiation genes [39]. Although BRG1/BRM and p63 are expressed in both progenitor and differentiation states, our data generation and analysis in this study only focused on differentiation day 4. It remains to be confirmed whether this cooperation also occurs in the epidermal progenitors to maintain the progenitor state.

The BAF complex has long been suggested to slide nucleosomes and to enhance TF binding to DNA, with early studies using in vitro DNase I cleavage experiments to probe changes on reconstituted chromatin templates in vitro [40, 41]. Using similar in vitro systems, it has also been indicated that transactivation domains, such as VP16, could facilitate BAF targeting to chromatin to stimulate its remodeling activity in vitro [42]. Very little has been determined, however, regarding how the BAF complex remodels genome-wide open chromatin landscape to regulate gene expression in vivo [43]. The present work indicates that BAF maintains DNA accessibility at ~11.6 % of open genomic regions in epidermal differentiation, with a strong selectivity towards lineage-specific regions rather than the common regions shared by multiple cell types. This suggests that the BAF complex does not function as a general chromatin remodeler, but displays strong selectivity in the context of developmental processes.

BAF has been demonstrated to associate with a variety of TFs from both in vitro and in vivo evidence [16, 25, 40, 41, 44, 45]. With the presence of hundreds of transcription regulators at a given developmental stage, it was unclear whether BAF displays any specificity in interacting selectively at binding sites of particular functionally active TFs to control open chromatin regions. Our data suggest that, during epidermal differentiation, the BAF complex exhibits strong specificity in cooperating with the lineage-determining TF p63 to activate gene expression by maintaining chromatin accessibility. Previous motif analysis indicated that a number of other TFs, such as RUNX1, RXRA, and TFAP2a, may function as co-activators for p63 [46]. This may explain the variations we observed for the accessibility changes in individual p63 sites with BAF knockdown. The co-binding of other TFs near p63 binding sites may make these sites more resistant to decreased p63 binding caused by BAF loss. In addition, it has been shown in mouse skin that p63 regulates Satb1 expression to further control chromatin architecture [47]. It would be of interest for future investigations to elucidate the crosstalk and the feedback regulation between chromatin modifiers and TFs to control gene expression in the context of tissue differentiation.

Our data also suggest that the BAF complex is required to maintain the accessibility at slightly more than 10 % of open chromatin regions genome-wide, at least in the context of epidermal differentiation. This raises the question of which factors maintain the open structure of the other ~90 %. One plausible possibility is that the other regions are controlled by the remodeling activity of other chromatin remodeling complexes, such as CHD, ISWI, and INO80 family members [48]. It also remains to be determined how much of the other open chromatin regions are pre-determined by their DNA sequence to dis-favor nucleosome formation.

We observed two well-positioned nucleosomes adjacent to p63 binding sites in normal epithelial differentiation. The role of nucleosome positioning in gene expression, while incompletely understood, has been best studied at TSSs. At the TSSs for active genes, a total of eight (three upstream and five downstream of the TSS) well-positioned nucleosomes are detected; however, only one +1 nucleosome is detected for silenced genes [49]. This suggests that well-positioned nucleosomes are positively correlated with gene expression. Much less is understood for intergenic DNA regions, including the distal regulatory regions observed here. Similar to what we have observed at p63 binding sites, well-positioned nucleosomes near TF binding sites have been reported at the Pu.1 sites in macrophages [50] and the androgen receptor sites in prostate cancer cells [51], although the basis for this positioning is unclear. Our data suggest that nucleosome phasing at regulatory regions requires the cooperation of a dominant TF, such as p63, and a chromatin remodeling complex, such as BAF, to overcome the intrinsic proclivity to nucleosome binding.

The BAF chromatin remodeling complex is a 2-MDa protein complex composed of 10 regulatory subunits and the interchangeable catalytic subunit BRG1 or BRM. The expression of BAF subunits differs in various cell types, and specific BAF subunits function to control specific gene expression [52]. To study the role of the BAF chromatin remodeling complex, we focused on its catalytic subunits. Taken together with the cooperativity and selectivity of BAF with p63 in epithelial tissue, it would be interesting to investigate the specific subunit composition of the BAF complex that interacts with p63. Furthermore, the BAF complex is frequently mutated in human cancers, including epidermal squamous cell carcinoma of the skin (SCC) [53]. Testing whether p63-BAF cooperativity in differentiation gene induction is compromised in SCC in a manner that inhibits tissue terminal differentiation and promotes cancer progression is also an intriguing future avenue of research. Given that p53 and p63 are structurally very similar to each other, it is also of future interest to investigate the degree of sustained cooperation between p53 and the BAF complex in both normal and cancer tissues.

## Conclusions

We demonstrate that the BAF chromatin remodeling complex plays an essential role in controlling the open chromatin landscape in somatic tissue differentiation. Although BAF binds broadly across the open chromatin landscape, BAF displays strong preferences in controlling the lineage-specific regions by cooperating with the master TF p63 in epidermal differentiation. These lineage-specific regions are characterized by a high intrinsic affinity to nucleosomes, and are inaccessible in other lineages. Maintenance of accessibility at the lineage-specific regions requires cooperation between BAF and the master TF to position the nucleosomes away from the TF recognition sites and to further recruit the transcriptional machinery to these regions, enabling tissue-specific gene expression.

## Methods

### Epidermal cell culture

Primary human epidermal keratinocytes were isolated from fresh surgically discarded skin from multiple donors. Keratinocytes from a minimum of three donors were mixed and cultured in complete Keratinocyte-SFM (Life Technologies, Grand Island, New York, USA,  #17005-142) and Medium 154 (Life Technologies #M-154-500). To induce terminal differentiation, keratinocytes were seeded in confluent condition with the addition of 1.2 mM Ca^2+^ in culture medium for 4 days. All sequencing experiments in this paper were carried out on differentiation day 4.

### On-plate ATAC-seq for differentiated keratinocytes

The on-plate ATAC-seq was modified based on the original ATAC-seq method [23] with the following modifications. First, 50,000 keratinocytes were seeded on a 96-well plate (Breiner Bio-one, Monroe, North Carolina, USA,  Cat# 675180). After inducing differentiation for 4 days, the cells were washed briefly with phosphate-buffered saline (PBS) two times, and permeabilized with lysis buffer (10 mM Tris-Cl pH 7.4, 10 mM NaCl, 3 mM MgCl_2_, 0.05 % NP-40) for 10 min. After this permeabilization step, the supernatant were removed, and replaced with 50 μL transposase and buffer mix (25 μL TD (2x reaction buffer from Nextera kit, Illumina, San Diego, California, USA), 5 μL TDE1(Tn5 Transposase from Nextera kit, Illumina), 20 μL nuclease-free H_2_O). After 30 min of incubation at 37 °C, a total volume of 250 μL Buffer PB (Qiagen, Hilden, Germany) was mixed with the reaction before DNA purification and polymerase chain reaction (PCR) enrichment. Each replicate of ATAC-seq data used in this study was generated individually using 50,000 keratinocytes growing in each well of a 96-well plate under differentiation conditions.

### Gene transfer and knockdown

For siRNA knockdown of BRG1/BRM, 1 × 10^6^ cells were electroporated with 1 nmol siRNA of each (ON-TARGETplus SMARTpool, Dharmacon, Lafayette, Colorado, USA) using Amaxa Human Keratinocyte Nucleofector Kit (Lonza, Basel, Switzerland, VPD-1002). To serve as control, 2 nmol total control siRNA were electroporated to 1 × 10^6^ cells. For siRNA knockdown of p63, an siRNA targeting pan p63 was used as published previously (sequence: CGACAGUCUUGUACAAUUUUU, Dharmacon) [32].

### Protein expression analysis

For immunoblot analysis, 20–50 μg of cell lysates were loaded per lane for sodium dodecyl sulfate polyacrylamide gel electrophoresis and transferred to polyvinylidene fluoride membranes. Primary antibodies were incubated at 4 °C overnight and secondary antibodies were incubated at room temperature for 1 h. The affinity purified J1 antiserum against BRG1/BRM was a generous gift of G Crabtree (Stanford University). Other antibodies used in this study include: anti-Krt1 (Covance, Princeton, New Jersey, USA), p63 (Santa Cruz Biotechnology, Santa Cruz, California, USA), p53 (Santa Cruz Biotechnology), and tubulin (Sigma, St. Louis, Missouri, USA).

### qRT-PCR expression analysis

For qRT-PCR, total RNA was extracted using the RNeasy Plus kit (Qiagen) and subsequently subjected to reverse transcription using the SuperScript VILO cDNA synthesis kit (Invitrogen). qRT-PCR (Quantitative Reverse Transcription Polymerase Chain Reaction) analysis was performed using the Mx3000P instrument with the SYBR Green Master Mix (Fermentas, Burlington, Ontario, Canada). Samples were run in duplicate and normalized to levels of GAPDH mRNA or 18S ribosomal RNA for each reaction.

### Chromatin immunoprecipitation

ChIP assays were performed essentially as previously described [28] with minor modifications. Human keratinocytes were cross-linked with 1 % formaldehyde at room temperature for 10 min. The chromatin was sonicated to achieve fragments with an average length of 200–500 bp. The sonicated chromatin was immunoprecipitated overnight at 4 °C with J1 antibody [29], Poll II (Millipore, Bedford, Massachusetts, USA), H3K27Ac (Active Motif, Calsbad, California, USA), H3K27me3 (Active Motif), H3K4me (Active Motif), or the same amount of IgG control (Cell Signaling). Following reverse cross-linking, the samples were treated with RNase and Protease K, and the DNA was purified using the Qiagen PCR Purification kit. For ChIP-seq, 10 ng of purified ChIP DNA were used to generate the sequencing library using NEBNext ChIP-Seq Library Prep Master Mix Set.

### Analysis of cell-type specificity of chromatin accessibility

Filtered reads from DHS-seq were downloaded as BAM files from the ENCODE project (http://hgdownload.cse.ucsc.edu/goldenPath/hg19/encodeDCC/wgEncodeUwDnase/). A set of universal DHS “clusters” identified by ENCODE was download from the UCSC Genome Browser (wgEncodeRegDnaseClusteredV3.bed), and DHS reads from each cell type were counted in each universal DHS region. DHS read counts were transformed with the arcsin function, and a z-score was calculated for the signal of each cell type for each region. Box plots were made using the R boxplot() function, and heatmaps were made using the heatmap.2 function in the R gplots package.

### Nucleosome probability analysis

Calculation of intrinsic nucleosome binding based on DNA sequence was performed using the Perl algorithm developed by Segal Lab [36]. Sequences of 6000 bp flanking the p63 motif sites were used as input genomic sequences. To remove the boundary effect, a window size of 4000 bp flanking the motif sites was used to plot the output. The same number and length of randomly shuttled genomic regions generated by Bedtools were used as a negative control for the analysis.

### Proximity ligation analysis

Primary human keratinocytes were cultured in differentiation condition on chamber slides, fixed by 4 % Formaldehyde Solution (Thermo Scientific, Rockford, Illinois, #28906) for 10 min at room temperature. Slides were blocked using blocking buffer (PBS with 0.3 % TritonX-100, 3 % normal horse serum) for 1 h at room temperature, followed by primary antibody incubation overnight at 4 °C. Slides were washed by PBS three times the next day, and were subsequently incubated with the PLA probes anti-mouse and anti-rabbit (Sigma). The Duolink In Situ Detection Reagents Orange (Sigma) was used for ligation and signal amplification.

### Nucleosome-free and mononucleosome ATAC-seq analysis

We first isolated paired-end ATAC-seq fragments from two length ranges: fragments shorter than 100 bp, and fragment lengths between 180 and 247 bp. These ranges were used previously [23] to distinguish transposase insertion into nucleosome-free DNA and insertions flanking nucleosomes, respectively. A 1-kb window centered on each region of interest was divided into ten 100 equal-sized bins. For each region, the number of fragment centers (single base pair) overlapping each bin was counted for each of the two fragment size ranges. The average fragment count plotted in each bin was normalized to the average fragment count in the first five bins in order to set the background signal to one. In order to facilitate comparisons across conditions in which ATAC-seq libraries exhibited markedly different signal to noise ratios, CTCF binding sites were used as a stable reference feature to scale average profiles. The enrichment profile was computed as described above for CTCF binding sites, and then a simple linear transformation was applied to match CTCF profiles across conditions. The same transformation was applied to profiles derived from other features of interest. For profiles derived from low-depth ATAC-seq libraries, a spline smoothing function from R (smooth.spline package) was used with a smoothing parameter between 0.3 and 0.5.

### V-plot analysis for nucleosome positioning

A volcano plot was constructed by plotting the normalized number of fragments. The length of the fragment was indicated by the y-axis; and the distance between the centers of the fragments to a p63 motif site was indicated by the x-axis. Volcano plots for both control and BAF loss were generated, enabling visual comparison of nucleosome positioned near p63 motif sites.

### ATAC-seq mapping, peak calls, and differential peak calls

ATAC-seq paired-end reads were trimmed for Illumina adapter sequences using a custom script and mapped to hg19 using bowtie with parameters –S –X2000 –m1. Duplicate reads were discarded with samtools. Peaks were identified using macs2 with parameters callpeak –nomodel –shift -100 –extsize 200. Peak sets called in individual replicates were combined and individual peaks merged if overlapping within 300 bp to form a union peak set. Differentially accessible peaks from this union set were identified with edgeR by counting all read ends overlapping peaks in each condition. edgeR was run with default settings, a fold-change threshold of 2, and FDR < 0.01.

### Single-nucleotide-resolution accessibility analysis

Because the Tn5 transposon binds as a dimer and inserts two adaptors separated by 9 bp [54], all reads aligning to the + strand were offset by +4 bp, and all reads aligning to the – strand were offset −5 bp. To predict the DNA binding of p63 and CTCF, we calculated the enrichment of the first base pair of each mapped and shifted ATAC-seq read in the motif sites within the TF binding sites from ChIP-seq analysis. p63 ChIP-seq in differentiated human keratinocytes [30] as well as the ENCODE CTCF ChIP-seq using human keratinocytes were used in this analysis.

### Cross cell type DHS analysis

DHS profiles were downloaded as BigWig files from the ENCODE portal at the UCSC genome browser (http://hgdownload.cse.ucsc.edu/goldenPath/hg19/encodeDCC/wgEncodeUwDnase/). The Homer annotatePeaks tool (http://homer.salk.edu/homer/) was used to calculate the average DHS signal in 10-bp bins surrounding p63 or CTCF sites.

### ChIP-seq metaplots

Metaplots of ChIP-seq signal (H3K27ac, H3K27me3, H3K4me1, RNA polymerase II, and BAF complex) were generated using the Homer annotatePeaks tool with reads counted in a 10-bp window and default library size normalization.

### RNA-seq library generation and data analysis

RNA extraction was performed using RNeasy Plus (Qiagen) from day 4 differentiated human keratinocytes treated with Brg1/Brm siRNA, p63 siRNA, or Control siRNA in technical replicates. rRNA was removed from each RNA extraction using Ribo-Zero rRNA Removal Kit (Illumina) before proceeding with RNA-seq library construction (NEBNext Ultra RNA Library Prep Kit, NEB). Paired-end sequencing of 2 × 75 bp was performed by Stanford Functional Genomics Facility using a Nextseq Sequencer (Illumina). Sequencing reads were mapped to hg19 using TopHat, raw read counts for each gene were calculated using an in-house generated perl script, and differential expression was analyzed using DEseq with the cutoff of FDR < 0.01, fold change >3.

### Data availability

Our ATAC-seq, ChIP-seq, and RNA-seq data sets have been deposited with the Gene Expression Omnibus [GEO: GSE67382]. The sequencing depth of these sequencing data is included in Additional file 2: Table S3.

### External datasets

The DHS data, RNA-seq data for 15 cell types as well as the CTCF ChIP-seq data in NHEK used in this study were generated by the ENCODE project. p63 ChIP-seq data in normal keratinocytes and the p63 transcriptome profiling analysis were extracted from previously published data sets generated by our group [30]. KLF4 motif sites within KLF4 ChIP-seq binding sites and RNA-seq data of differentiated human keratinocytes were also extracted from previously published studies from our group [55, 56].

### Ethics

This study was conducted under IRB-35324 (Stanford University). The keratinocytes used in this study were isolated from surgically discarded foreskin before January 2015, and were mixed from 3–5 de-identified donors. The tissue donation was under IRB exempt protocol at Stanford. We confirm that the experimental methods comply with the Helsinki Declaration.

## Additional files

Additional file 1:
**Includes the supplementary Figures S1–4.** **Figure S1**. BAF is essential for epidermal gene induction. (**a**) Quantitative RT-PCR analysis demonstrating the knockdown efficiency of BRG1/BRM siRNAs and the suppression of differentiation gene expression in BAF loss compared to control. (**b**) GO analysis of the significant changed genes (fold change > 3, FDR < 0.01) with BAF knockdown in RNA-seq. (**c**) Western blot analysis showing the time course of 4-day differentiation induction in primary human keratinocytes, comparing BRG1/BRM loss with control. BRG1/BRM siRNA efficiently knocked down BRG1 and BRM protein levels. The induction of Keratin 1 is significantly impaired with BAF loss, although no significant changes were detected with p63 and p53 protein level relative to tubulin loading control. (**d**–**f**) Distribution of total ATAC-seq peaks, and the BAF-dependent ATAC-seq peaks relative to gene promoter, exon, intron and distal regulatory regions. **Figure S2**. BAF maintains open chromatin regions with p63 binding sites. (**a**) Scatter plot demonstrating correlation between BAF binding and open chromatin across the genome. (**b**) RNA expression levels (RPKM) of p53 family transcription factors in human keratinocytes. (**c**) Pie chart demonstrating the percentage of p63 motif sites at p63 binding sites that became inaccessible with BAF knockdown. (**d**) RNA expression levels of all expressed TFs in keratinocytes. Gray dots indicate the expression levels of 809 TFs (RPKM > 1 in differentiating human keratinocytes) listed in GO. Representative TFs known to be functional in epidermal differentiation along with CTCF are highlighted in brown and red. (**e**) ATAC-seq accessibility in KLF4 motif sites in KLF4 binding sites comparing control vs BAF loss conditions. (**f**) Heatmap showing the fold changes of the shared 236 genes (fold change > 3, FDR < 0.01) controlled by both BAF and p63. (**g**) Representative RNA-seq data tracks of BAFi, p63i, and CTRLi replicates. **Figure S3**. BAF loss does not affect nucleosome positioning or genome accessibility at CTCF binding regions. (**a**) ATAC-seq fragment size distribution. Gray shaded area represents nucleosome-free fragments (<100 bp), and blue shaded area represents mononucleosome fragments (180–247 bp). Schematic illustration of these ATAC-seq fragments is shown on the right. (**b**, **c**) V-plot analysis demonstrating the nucleosome positioning at CTCF motif regions comparing control versus BAF loss. (**d**) Average diagram of nucleosome-free ATAC-seq fragments at CTCF motif regions comparing control and BAF loss. (**e**) Average diagram of mononucleosome ATAC-seq fragments at CTCF motif regions comparing control and BAF loss. (**f**) Average diagram of predicted nucleosome binding probability based on DNA sequences using same number of shuffled genomic regions as in p63 motif nucleosome probability analysis. **Figure S4**. BAF loss impairs p63 binding to its target sites. (**a**, **b**) Single nucleotide ATAC accessibility analysis with 1-bp resolution at p63 and CTCF motif regions in their ChIP-seq binding sites, comparing control and BAF loss. (**c**) Summit-centered heatmap comparing p63 ChIP-seq peaks in control and BAF loss. (**d**, **e**) Average diagram of p63 ChIP-seq signal enrichment in the peaks that are overlapped or unique in control. (**f**) Average diagram of BAF ChIP-seq signal at CTCF sites comparing p63 loss with control conditions. (PDF 3349 kb) Additional file 2: Table S1. Genes changed >3-fold with BRG1/BRM knockdown. **Table S2** Shared genes controlled by both BAF and p63 with fold change > 3. **Table S3** Sequencing depth of the ATAC-seq, RNA-seq, and ChIP-seq data generated for this study. (XLSX 78 kb)

## Abbreviations

ATAC-seq: Assay for transposase-accessible chromatin with high throughput sequencing
BAF: Brg/Brm associated factor
bp: base pair
ChIP-seq: chromatin immunoprecipitation followed by high-throughput DNA sequencing
DHS: DNaseI hypersensitivity site
ENCODE: Encyclopedia of DNA Elements
FAIRE-seq: formaldehyde-assisted isolation of regulatory elements followed by high-throughput sequencing
FDR: false discovery rate
GO: Gene Ontology
NHEK: normal human epidermal keratinocytes
PBS: phosphate-buffered saline
PCR: polymerase chain reaction
PLA: proximity ligation analysis
SCC: squamous cell carcinoma
TF: transcription factor
TSS: transcription start site

## References

[1] Agarwal AK, Garg A (2006). Genetic disorders of adipose tissue development, differentiation, and death. Annu Rev Genomics Hum Genet..

[2] Thelu J, Rossio P, Favier B (2002). Notch signalling is linked to epidermal cell differentiation level in basal cell carcinoma, psoriasis and wound healing. BMC Dermatol..

[3] Reuter JA, Ortiz-Urda S, Kretz M, Garcia J, Scholl FA, Pasmooij AMG (2009). Modeling inducible human tissue neoplasia identifies an extracellular matrix interaction network involved in cancer progression. Cancer Cell..

[4] Ridky TW, Chow JM, Wong DJ, Khavari PA (2010). Invasive three-dimensional organotypic neoplasia from multiple normal human epithelia. Nat Med..

[5] Stergachis AB, Neph S, Reynolds A, Humbert R, Miller B, Paige SL (2013). Developmental fate and cellular maturity encoded in human regulatory DNA landscapes. Cell..

[6] Natarajan A, Yardimci GG, Sheffield NC, Crawford GE, Ohler U (2012). Predicting cell-type-specific gene expression from regions of open chromatin. Genome Res..

[7] Song L, Zhang Z, Grasfeder LL, Boyle AP, Giresi PG, Lee B-K (2011). Open chromatin defined by DNaseI and FAIRE identifies regulatory elements that shape cell-type identity. Genome Res..

[8] Boyle AP, Davis S, Shulha HP, Meltzer P, Margulies EH, Weng Z (2008). High-resolution mapping and characterization of open chromatin across the genome. Cell..

[9] Giresi PG, Kim J, McDaniell RM, Iyer VR, Lieb JD (2007). FAIRE (Formaldehyde-Assisted Isolation of Regulatory Elements) isolates active regulatory elements from human chromatin. Genome Res..

[10] Hang CT, Yang J, Han P, Cheng HL, Shang C, Ashley E, et al. Chromatin regulation by Brg1 underlies heart muscle development and disease. Nature. 2010; 466:62-67. 10.1038/nature09130 PMC2898892 20596014

[11] De la Serna IL, Carlson KA, Imbalzano AN (2001). Mammalian SWI/SNF complexes promote MyoD-mediated muscle differentiation. Nat Genet..

[12] Indra AK, Dupé V, Bornert J-M, Messaddeq N, Yaniv M, Mark M (2005). Temporally controlled targeted somatic mutagenesis in embryonic surface ectoderm and fetal epidermal keratinocytes unveils two distinct developmental functions of BRG1 in limb morphogenesis and skin barrier formation. Development..

[13] Bao X, Tang J, Lopez-Pajares V, Tao S, Qu K, Crabtree GR (2013). ACTL6a enforces the epidermal progenitor state by suppressing SWI/SNF-dependent induction of KLF4. Cell Stem Cell..

[14] Hu G, Schones DE, Cui K, Ybarra R, Northrup D, Tang Q (2011). Regulation of nucleosome landscape and transcription factor targeting at tissue-specific enhancers by BRG1. Genome Res..

[15] Gresh L, Bourachot B, Reimann A, Guigas B, Fiette L, Garbay S (2005). The SWI/SNF chromatin-remodeling complex subunit SNF5 is essential for hepatocyte differentiation. EMBO J..

[16] Kadoch C, Hargreaves DC, Hodges C, Elias L, Ho L, Ranish J (2013). Proteomic and bioinformatic analysis of mammalian SWI/SNF complexes identifies extensive roles in human malignancy. Nat Genet..

[17] Wilson BG, Roberts CWM (2011). SWI/SNF nucleosome remodellers and cancer. Nat Rev Cancer..

[18] Utley RT (1997). SWI/SNF Stimulates the formation of disparate activator-nucleosome complexes but is partially redundant with cooperative binding. J Biol Chem..

[19] De la Serna IL, Ohkawa Y, Berkes CA, Bergstrom DA, Dacwag CS, Tapscott SJ (2005). MyoD targets chromatin remodeling complexes to the myogenin locus prior to forming a stable DNA-bound complex. Mol Cell Biol..

[20] Morris SA, Baek S, Sung M-H, John S, Wiench M, Johnson TA (2014). Overlapping chromatin-remodeling systems collaborate genome wide at dynamic chromatin transitions. Nat Struct Mol Biol..

[21] Moshkin YM, Mohrmann L, van Ijcken WFJ, Verrijzer CP (2007). Functional differentiation of SWI/SNF remodelers in transcription and cell cycle control. Mol Cell Biol..

[22] Willis MS, Homeister JW, Rosson GB, Annayev Y, Holley D, Holly SP (2012). Functional redundancy of SWI/SNF catalytic subunits in maintaining vascular endothelial cells in the adult heart. Circ Res..

[23] Buenrostro JD, Giresi PG, Zaba LC, Chang HY, Greenleaf WJ (2013). Transposition of native chromatin for fast and sensitive epigenomic profiling of open chromatin, DNA-binding proteins and nucleosome position. Nat Methods..

[24] Huang H, Hsing H, Lai T, Chen Y, Lee T, Chan H (2010). Trypsin-induced proteome alteration during cell subculture in mammalian cells. J Biomed Sci..

[25] Banno T, Blumenberg M (2014). Keratinocyte detachment-differentiation connection revisited, or Anoikis-Pityriasi Nexus Redux. PLoS One..

[26] Sethi I, Sinha S, Buck MJ (2014). Role of chromatin and transcriptional co-regulators in mediating p63-genome interactions in keratinocytes. BMC Genomics..

[27] Ho L, Jothi R, Ronan JL, Cui K, Zhao K, Crabtree GR (2009). An embryonic stem cell chromatin remodeling complex, esBAF, is an essential component of the core pluripotency transcriptional network. Proc Natl Acad Sci U S A..

[28] Euskirchen GM, Auerbach RK, Davidov E, Gianoulis TA, Zhong G, Rozowsky J (2011). Diverse roles and interactions of the SWI/SNF chromatin remodeling complex revealed using global approaches. PLoS Genet..

[29] Khavari PA, Peterson CL, Tamkun JW, Mendel DB, Crabtree GR (1993). BRG1 contains a conserved domain of the SWI2/SNF2 family necessary for normal mitotic growth and transcription. Nature..

[30] Zarnegar BJ, Webster DE, Lopez-Pajares V, Vander Stoep Hunt B, Qu K, Yan KJ (2012). Genomic profiling of a human organotypic model of AEC syndrome reveals ZNF750 as an essential downstream target of mutant TP63. Am J Hum Genet.

[31] Smeenk L, van Heeringen SJ, Koeppel M, van Driel MA, Bartels SJJ, Akkers RC (2008). Characterization of genome-wide p53-binding sites upon stress response. Nucleic Acids Res..

[32] Truong AB, Kretz M, Ridky TW, Kimmel R, Khavari PA (2006). p63 regulates proliferation and differentiation of developmentally mature keratinocytes. Genes Dev..

[33] Mills AA, Zheng B, Wang XJ, Vogel H, Roop DR, Bradley A (1999). p63 is a p53 homologue required for limb and epidermal morphogenesis. Nature..

[34] Armstrong JA, Bieker JJ, Emerson BM (1998). A SWI/SNF–related chromatin remodeling complex, E-RC1, is required for tissue-specific transcriptional regulation by EKLF in vitro. Cell..

[35] Martens JA, Winston F (2003). Recent advances in understanding chromatin remodeling by Swi/Snf complexes. Curr Opin Genet Dev..

[36] Kaplan N, Moore IK, Fondufe-Mittendorf Y, Gossett AJ, Tillo D, Field Y (2009). The DNA-encoded nucleosome organization of a eukaryotic genome. Nature..

[37] Segal E, Fondufe-Mittendorf Y, Chen L, Thåström A, Field Y, Moore IK (2006). A genomic code for nucleosome positioning. Nature..

[38] Kowenz-Leutz E, Leutz A (1999). A C/EBPβ isoform recruits the SWI/SNF complex to activate myeloid genes. Mol Cell..

[39] Lopez-Pajares V, Qu K, Zhang J, Webster DE, Barajas BC, Siprashvili Z (2015). A LncRNA-MAF:MAFB transcription factor network regulates epidermal differentiation. Dev Cell..

[40] Cote J, Peterson CL, Workman JL (1998). Perturbation of nucleosome core structure by the SWI/SNF complex persists after its detachment, enhancing subsequent transcription factor binding. Proc Natl Acad Sci..

[41] Kwon H, Imbalzano AN, Khavari PA, Kingston RE, Green MR (1994). Nucleosome disruption and enhancement of activator binding by a human SW1/SNF complex. Nature..

[42] Gutiérrez JL, Chandy M, Carrozza MJ, Workman JL (2007). Activation domains drive nucleosome eviction by SWI/SNF. EMBO J..

[43] Hargreaves DC, Crabtree GR (2011). ATP-dependent chromatin remodeling: genetics, genomics and mechanisms. Cell Res..

[44] Seo S, Richardson GA, Kroll KL (2005). The SWI/SNF chromatin remodeling protein Brg1 is required for vertebrate neurogenesis and mediates transactivation of Ngn and NeuroD. Development..

[45] Pedersen TA, Kowenz-Leutz E, Leutz A, Nerlov C (2001). Cooperation between C/EBPalpha TBP/TFIIB and SWI/SNF recruiting domains is required for adipocyte differentiation. Genes Dev..

[46] Kouwenhoven EN, Oti M, Niehues H, van Heeringen SJ, Schalkwijk J, Stunnenberg HG (2015). Transcription factor p63 bookmarks and regulates dynamic enhancers during epidermal differentiation. EMBO Rep..

[47] Fessing MY, Mardaryev AN, Gdula MR, Sharov AA, Sharova TY, Rapisarda V (2011). p63 regulates Satb1 to control tissue-specific chromatin remodeling during development of the epidermis. J Cell Biol..

[48] Clapier CR, Cairns BR. The biology of chromatin remodeling complexes. Annu Rev Biochem. 2009;78:273-304. 10.1146/annurev.biochem.77.062706.153223 19355820

[49] Schones DE, Cui K, Cuddapah S, Roh T-Y, Barski A, Wang Z (2008). Dynamic regulation of nucleosome positioning in the human genome. Cell..

[50] Barozzi I, Simonatto M, Bonifacio S, Yang L, Rohs R, Ghisletti S (2014). Coregulation of transcription factor binding and nucleosome occupancy through DNA features of mammalian enhancers. Mol Cell..

[51] He HH, Meyer CA, Shin H, Bailey ST, Wei G, Wang Q (2010). Nucleosome dynamics define transcriptional enhancers. Nat Genet..

[52] Wu JI, Lessard J, Crabtree GR (2009). Understanding the words of chromatin regulation. Cell..

[53] Lee CS, Bhaduri A, Mah A, Johnson WL, Ungewickell A, Aros CJ (2014). Recurrent point mutations in the kinetochore gene KNSTRN in cutaneous squamous cell carcinoma. Nat Genet..

[54] Adey A, Morrison HG, Asan, Xun X, Kitzman JO, Turner EH (2010). Rapid, low-input, low-bias construction of shotgun fragment libraries by high-density in vitro transposition. Genome Biol..

[55] Boxer LD, Barajas B, Tao S, Zhang J, Khavari PA (2014). ZNF750 interacts with KLF4 and RCOR1, KDM1A, and CTBP1/2 chromatin regulators to repress epidermal progenitor genes and induce differentiation genes. Genes Dev..

[56] Kretz M, Webster DE, Flockhart RJ, Lee CS, Zehnder A, Lopez-Pajares V (2012). Suppression of progenitor differentiation requires the long noncoding RNA ANCR. Genes Dev..

