# Supplementary material for: A novel ATAC-seq approach reveals lineage-specific reinforcement of the open chromatin landscape via cooperation between BAF and p63
Source: Genome Biol. 2015 Dec 18;16:284. doi: 10.1186/s13059-015-0840-9 (PMC4699366; doi:10.1186/s13059-015-0840-9)

Figure S1. BAF is essential for epidermal differentiation gene induction

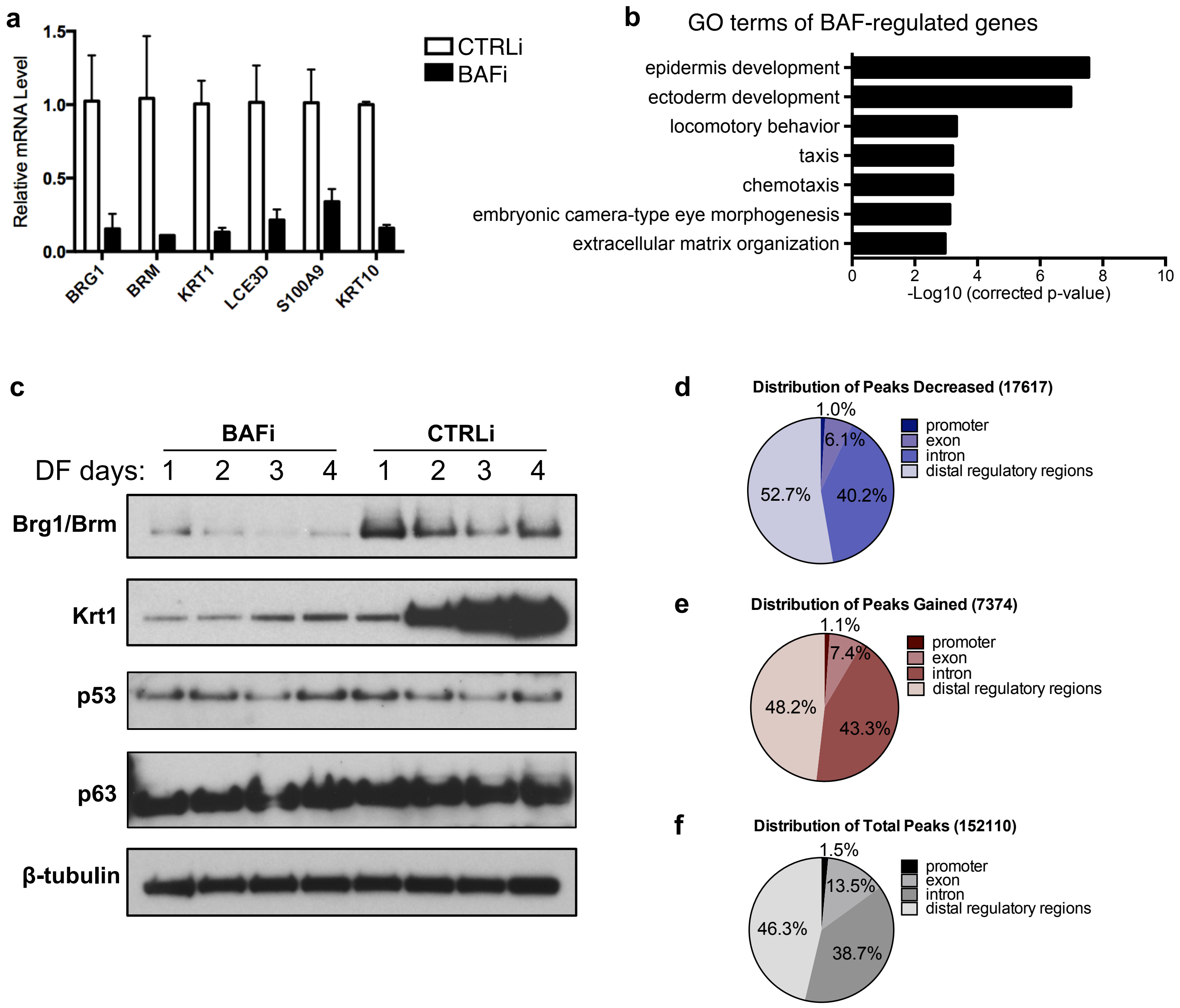

Figure S2. BAF maintains open chromatin regions with p63 binding sites

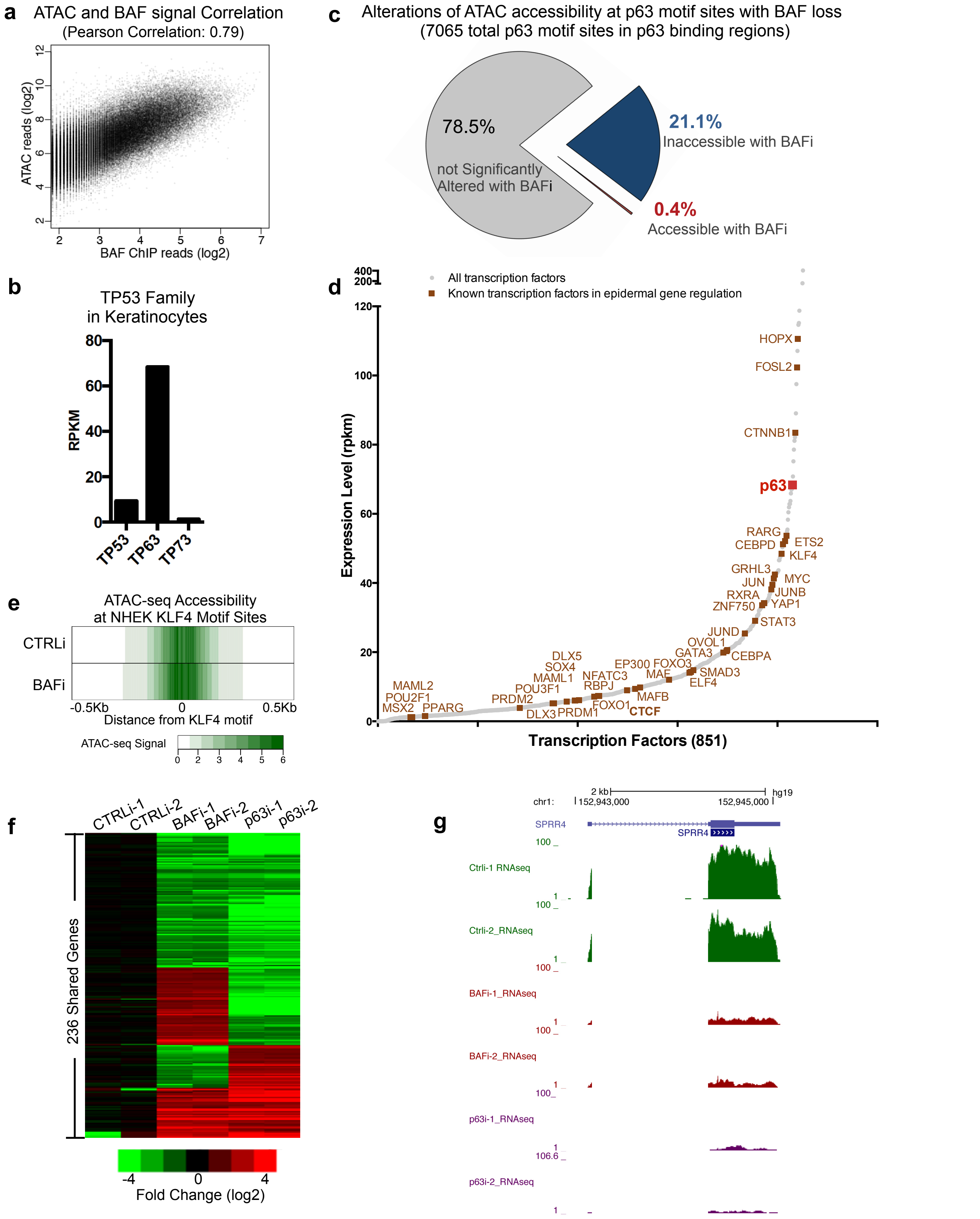

Figure S3. BAF loss does not affect nucleosome positioning or accessibility at CTCF sites

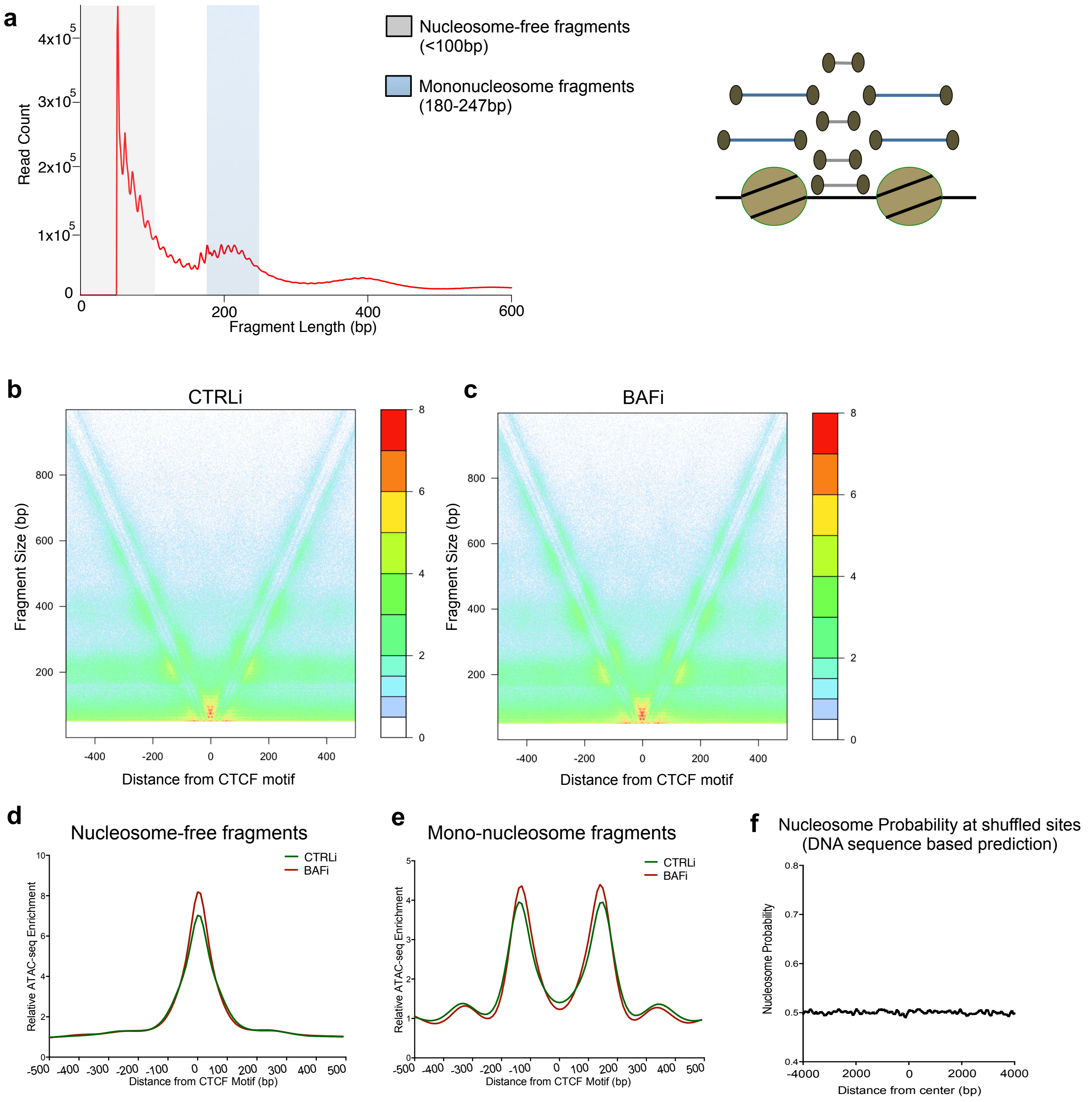

Figure S4. BAF loss impairs p63 binding to its target sites

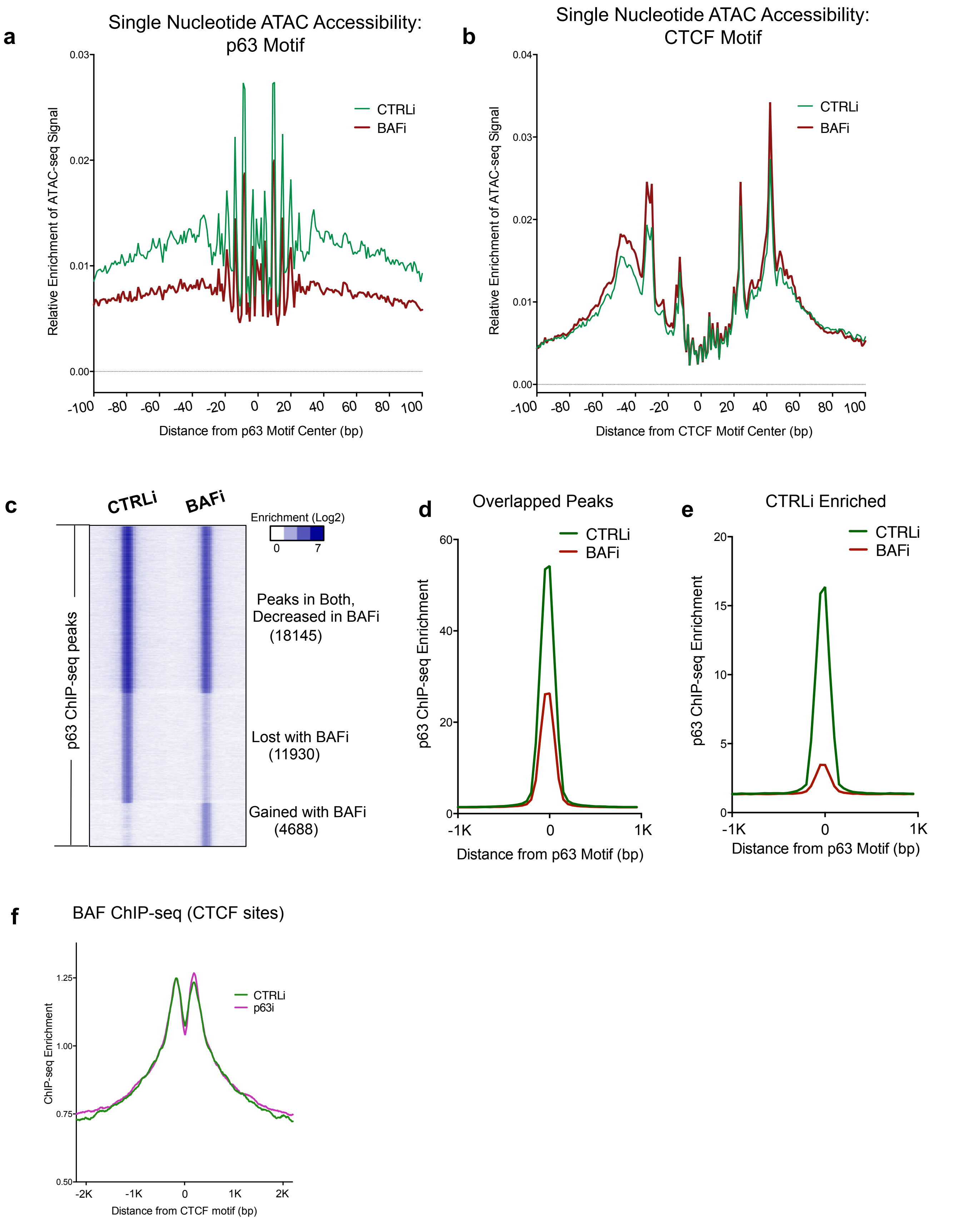

Supplement: Additional file 1: — Includes the supplementary Figures S1–4. Figure S1. BAF is essential for epidermal gene induction. (a) Quantitative RT-PCR analysis demonstrating the knockdown efficiency of BRG1/BRM siRNAs and the suppression of differentiation gene expression in BAF loss compared to control. (b) GO analysis of the significant changed genes (fold change > 3, FDR < 0.01) with BAF knockdown in RNA-seq. (c) Western blot analysis showing the time course of 4-day differentiation induction in primary human keratinocytes, comparing BRG1/BRM loss with control. BRG1/BRM siRNA efficiently knocked down BRG1 and BRM protein levels. The induction of Keratin 1 is significantly impaired with BAF loss, although no significant changes were detected with p63 and p53 protein level relative to tubulin loading control. (d–f) Distribution of total ATAC-seq peaks, and the BAF-dependent ATAC-seq peaks relative to gene promoter, exon, intron and distal regulatory regions. Figure S2. BAF maintains open chromatin regions with p63 binding sites. (a) Scatter plot demonstrating correlation between BAF binding and open chromatin across the genome. (b) RNA expression levels (RPKM) of p53 family transcription factors in human keratinocytes. (c) Pie chart demonstrating the percentage of p63 motif sites at p63 binding sites that became inaccessible with BAF knockdown. (d) RNA expression levels of all expressed TFs in keratinocytes. Gray dots indicate the expression levels of 809 TFs (RPKM > 1 in differentiating human keratinocytes) listed in GO. Representative TFs known to be functional in epidermal differentiation along with CTCF are highlighted in brown and red. (e) ATAC-seq accessibility in KLF4 motif sites in KLF4 binding sites comparing control vs BAF loss conditions. (f) Heatmap showing the fold changes of the shared 236 genes (fold change > 3, FDR < 0.01) controlled by both BAF and p63. (g) Representative RNA-seq data tracks of BAFi, p63i, and CTRLi replicates. Figure S3. BAF loss does not affect nucleosome p [file 13059_2015_840_MOESM1_ESM.pdf]
